# Exceptional reactivity of the bridgehead amine on bicyclo[1.1.1]pentane

**DOI:** 10.24820/ark.5550190.p012.003

**Published:** 2023-08-23

**Authors:** Yong Lu, Chuo Chen

**Affiliations:** Department of Biochemistry, UT Southwestern Medical Center, 5323 Harry Hines Boulevard, Dallas, TX 75390-9038

**Keywords:** Bicyclo[1.1.1]pentane (BCP), bridgehead amine, steric hindrance, intrinsic nucleophilicity

## Abstract

Bicyclo[1.1.1]pentane (BCP) has received substantial interest in the field of synthetic chemistry recently for its potential use as a benzene isostere in medicinal chemistry. Whereas bicyclo[2.2.2]octane (BCO) has also been used as a bioisostere of benzene, the condensation of BCP-amine with nadic anhydride is significantly easier than that of BCO-amine. Analyses of the geometries and the frontier molecular orbitals of these amines suggest that the low steric hindrance and high intrinsic nucleophilicity of BCP-amine together contribute to its exceptional reactivity.

## Graphical Abstract

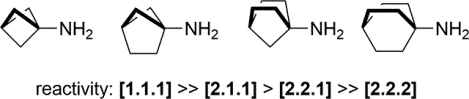

## Introduction

Bicyclo[1.1.1]pentane (BCP) was initially prepared as a synthetic exercise of a structurally unique, strained ring system to understand its chemical reactivity and physical properties.^1–11^ Recently, it has received substantial interest in the field of synthetic and medicinal chemistry due to its potential use as a benzene isostere with enhanced aqueous solubility and metabolic stability.^12–15^ Various new synthetic approaches have been developed to improve its accessibility.^16–19^ Similar to bicyclo[2.2.2]octane (BCO),^20–23^ the fully sp^3^-hybridized BCP is a hydrophobic group ideal for filling enzyme pockets wherein π-π interaction is not needed. We sought to explore the possibility of using BCP or BCO to improve the drug properties of IWR1 (**1**), a highly selective tankyrase inhibitor (TNKSi)^24^ that binds to the adenosine (AD) site of tankyrase (TNKS) to induce a D-loop conformation change of this unique PARP family protein to prevent poly(ADP-ribosylation) of its substrates.^25^ However, **1** has low aqueous solubility and poor metabolic stability.^26^ During the synthesis of the BCP and BCO analogs of **1**, we found that the BCP-amine reacted with nadic anhydride easily but the BCO-amine sluggishly. Analyses of the geometries and the frontier molecular orbitals of these amines provided an explanation to the reactivities of these structurally unique primary amines. A combination of low steric hindrance and high intrinsic nucleophilicity of the BCP-amine contributes to its exceptional reactivity.

## Results and Discussion

**Figure 1.**
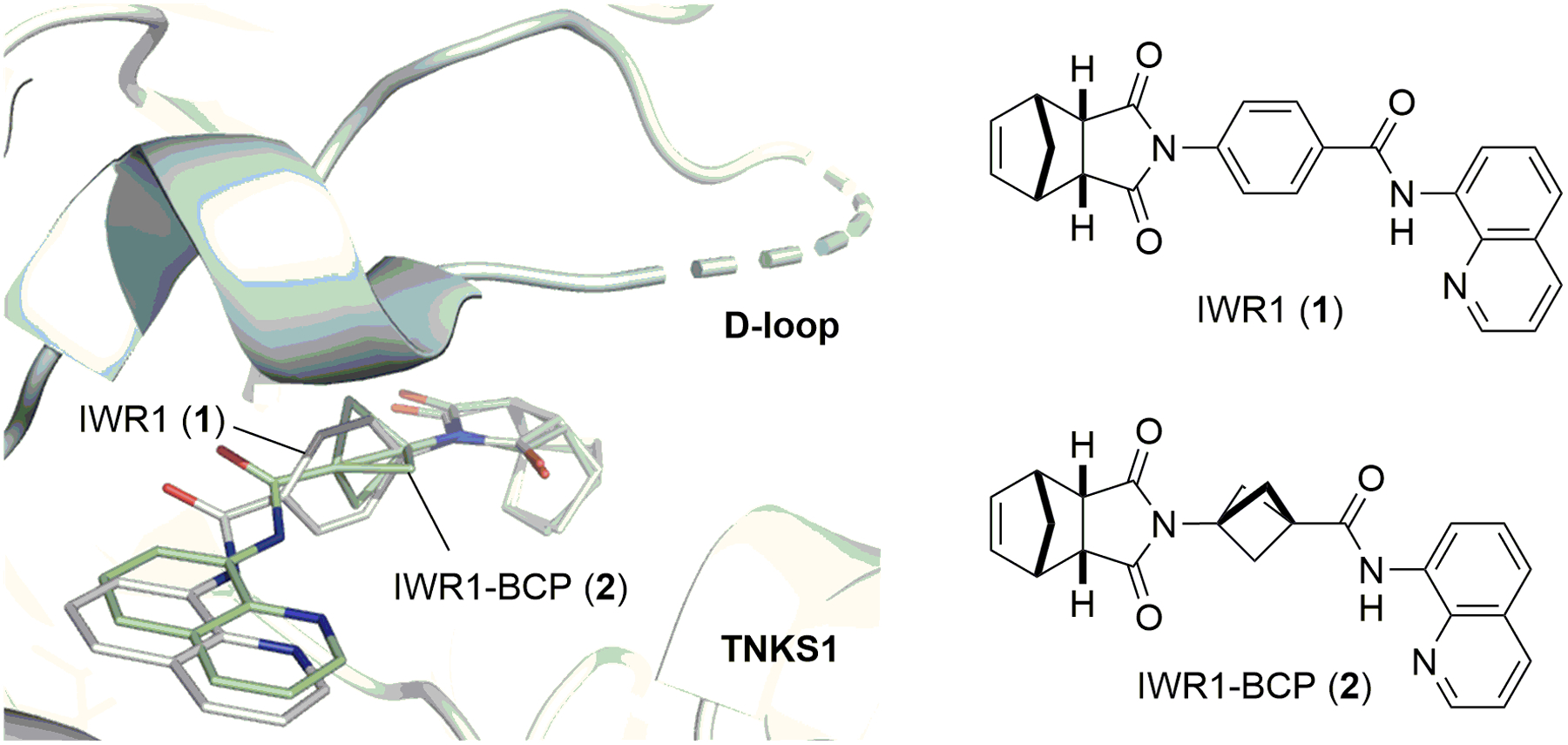
The TNKS-binding mode of **1** and **2** predicted by Autodock Vina.

**Figure 2.**
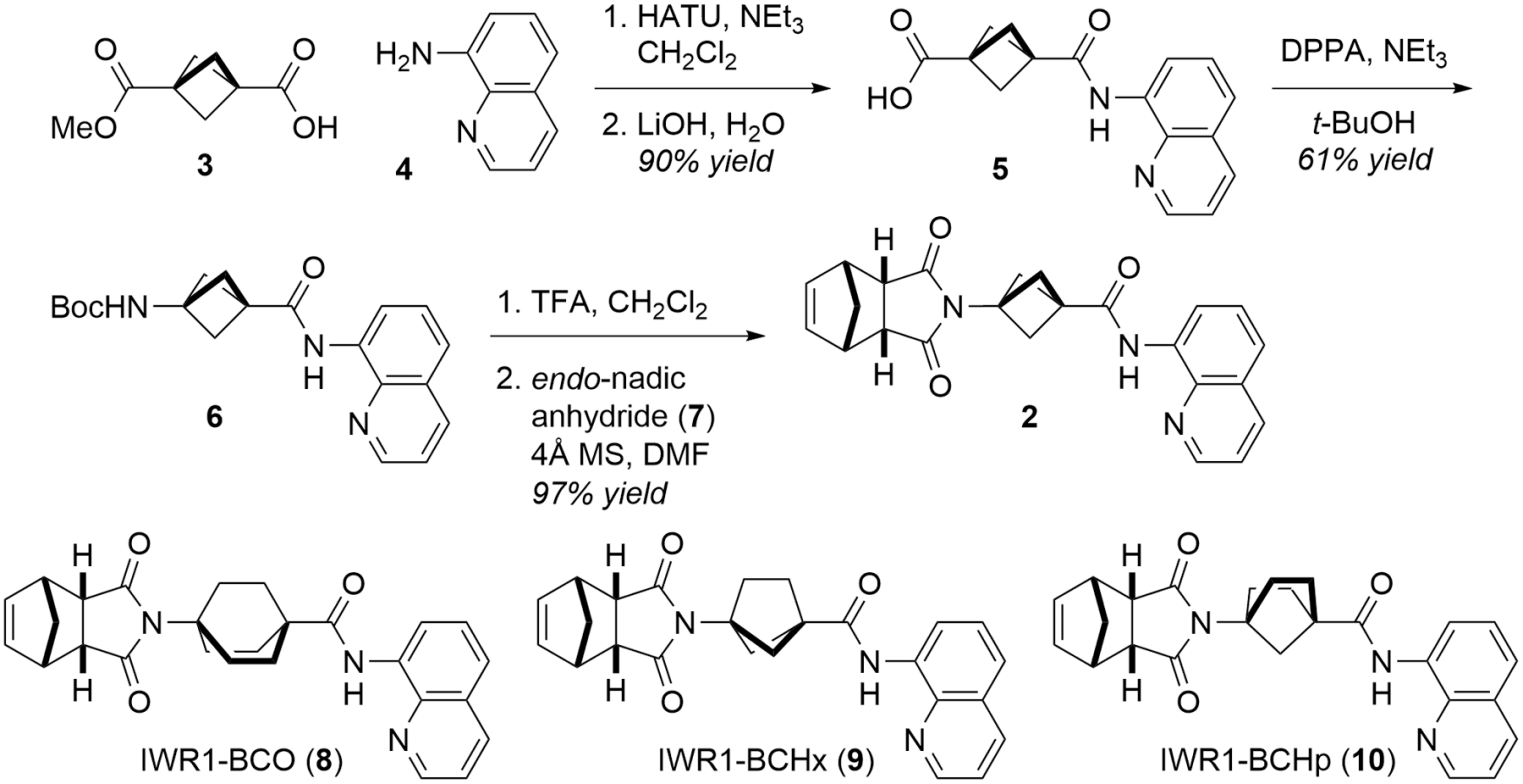
The synthesis of **2** and the structures of **8**‒**10**.

1,3-Disubstituted BCP is a bioisostere of 1,4-disubstituted benzene. Docking experiments suggest that substituting the benzene ring of **1** with BCP would not affect its affinity to TNKS (Figure 1). We thus prepared IWR1-BCP (**2**) using a simple 5-step procedure (Figure 2). Coupling BCP-acid **3** with 8-aminoquinoline (**4**) followed by hydrolyzing its ester group gave **5**. Subsequent Curtius rearrangement in *tert*-butanol yielded **6**, a carbamate that could also be prepared directly from coupling the more expensive BocNH-BCP-COOH with **4**. After removing the Boc group, condensing the resulting amine with *endo*-nadic anhydride (**7**) afforded **2** with a good overall yield. To further evaluate the steric tolerance of the AD pocket of TNKS wherein the benzene ring of **1** sits, we prepared IWR1-BCO (**8**) in a similar manner as 1,3-disubstituted BCO is also a commonly used 1,4-benzene bioisostere.^27,28^ However, the norendimide condensation proceeded with a poor yield. To understand the observed reactivity difference, we also synthesized IWR1-BCHx (**9**) and IWR1-BCHp (**10**) analogously and found that the condensation efficiency gradually decreased as the size of the bicyclic ring system increases (BCP-**2**: 97% → BCHx-**9**: 49% → BCHp-**10**: 37% → BCO-**8**: 7%).

**Figure 3.**
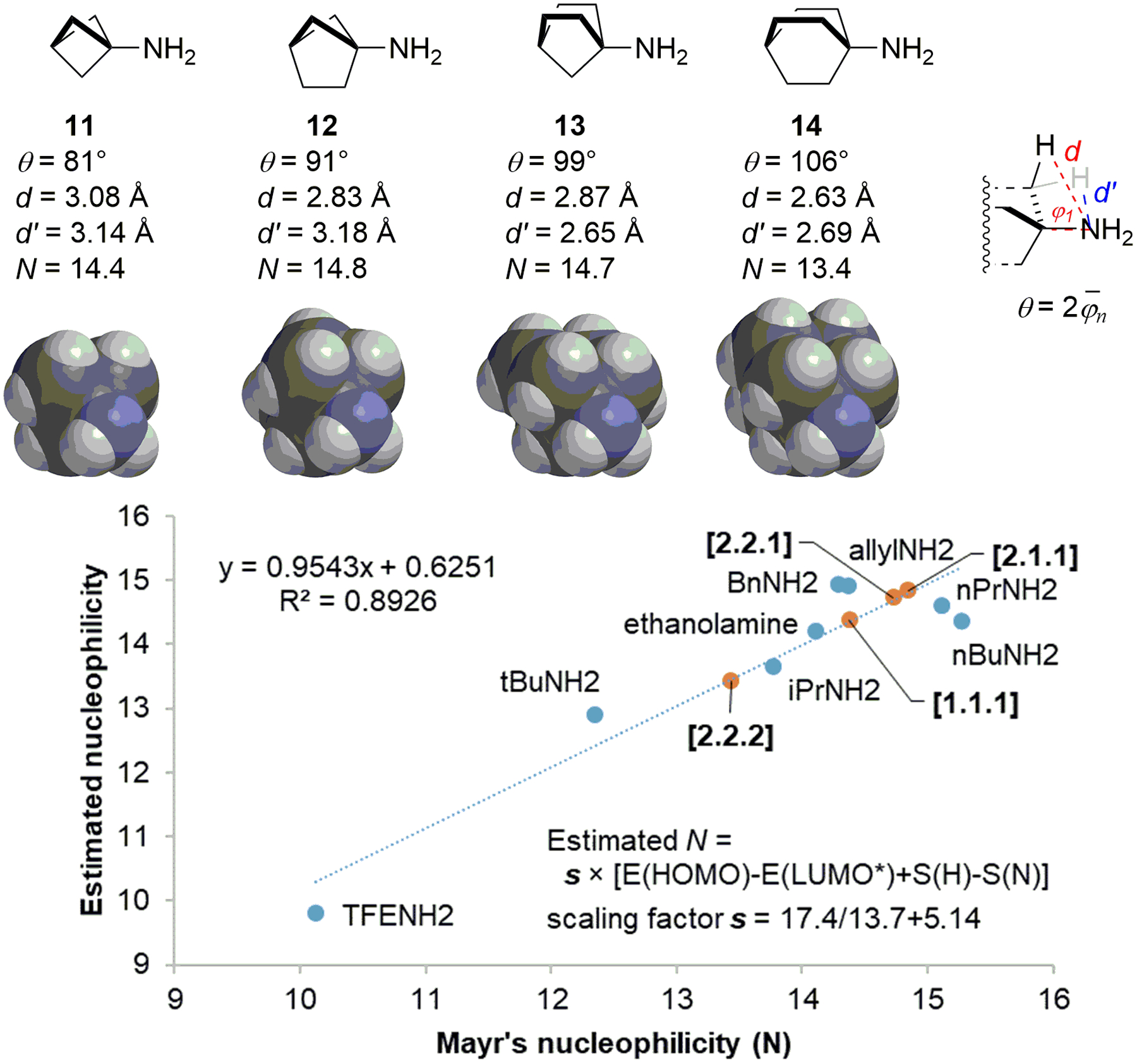
The stereoelectronic factors of **11**‒**14**.

To probe the dramatic difference in the condensation efficiency of these bicyclic amines, we assessed the nucleophilicity of **11**─**14** computationally to determine the contribution of the stereoelectronic effects of the ring system to the reactivity of the attached primary amine. We first calculated the equilibrium geometries of 8 primary amines (*n*-propyl, *n*-butyl, allyl, benzyl, 2-hydroxyethyl, *i*-propyl, *t*-butyl, and trifluoroethyl) and the corresponding ammonium ions by DFT at the B3LYP/6–311G+(d,p) level. We then used their HOMO and LUMO energies to evaluate various methods of nucleophilicity prediction.^29,30^ We found that the energy difference between the HOMO of the amine and the LUMO of the ammonium ion correlates well with the experimental data obtained in acetonitrile,^31^ and the single-point energies calculated with the 6–311G+(d,p) basis set provided significantly better prediction than def2-TZVP. Adding the SMD solvation energy calculated at the 6–31G(d) level further improved the correlation (Figure 3). We thus used this model to estimate the intrinsic nucleophilicity of **11**─**14**. Interestingly, introducing ring strains increases the s-character of the C─N bond (**14**: 27% → **13**: 28% → **12**: 30% → **11**: 34%)^32^ but does not attenuate their nucleophilicity. Amines **11**─**14** are all predicted to be more nucleophilic than aniline (*N* = 12.64). In particular, **11** has significantly higher nucleophilicity than aniline despite nearly sp^2^-hybridized at the bridgehead position. However, **11** is predicted to have lower nucleophilicity than **12** and **13**. The nucleophilicities of **12** and **13** are expected to be close to that of *n*-propylamine, **11** to be equivalent to allylamine and benzylamine, and **14** to reside between *iso-*propylamine and *tert*-butylamine.

To further understand the exceptional reactivity of BCP-amine, we assessed the steric hindrance of **11**─**14** by calculating the cone angles of these amines. As expected, the cone angle gradually increases as the ring size increases, with **12** close to *iso*-propylamine (90°) and **14** close to *tert*-butylamine (106°). Moreover, the distance between the nitrogen atom and the nearest hydrogen atom that blocks the approach of the electrophile is the longest for BCP and the shortest for BCO. This rather subtle steric effect apparently further tunes the reactivity of the amino group attached to the bridgehead position of these bicyclic systems. As such, the unique size of BCP-amine granted it exceptional reactivity toward nucleophilic reactions.

## Conclusions

The nucleophilicity of a series of primary amines can be approximated by the energy difference between the HOMO of the amine and the LUMO of its ammonium ion, and this prediction can further be improved by including solvation energies. Despite nearly sp^2^-hybridized at the bridgehead position, **11** is expected to be significantly more nucleophilic than aniline. Additionally, the difference in the norendimide condensation efficiency of **2**, **8**, **9**, and **10** cannot be explained simply by the electronic properties of these primary amines. A combination of low steric hindrance and high intrinsic nucleophilicity of the BCP-amine contributes to its exceptional reactivity.

## Experimental Section

### General.

All solvents for the synthesis were purified by passing commercially available pre-dried, oxygen-free formulations through activated alumina columns. Reactions were monitored by TLC or LC-MS and the products were purified by flash column chromatography unless otherwise mentioned. NMR spectra were recorded on a Bruker AN400 or AN600 instrument. The chemical shifts for ^1^H and ^13^C NMR spectra are reported in ppm (δ) relative to the ^1^H and ^13^C signals in the solvent (CDCl_3_: δ 7.26, 77.16 ppm; CD_3_CN: δ 1.94, 118.26 ppm; CD_3_OD: δ 3.31, 49.00 ppm) and the multiplicities are presented as follows: s = singlet, d = doublet, t = triplet, m = multiplet. LC-MS was performed on an Agilent 1260 HPLC machine coupled to a 6120 single quadrupole MS detector using an Agilent Eclipse XDB-C18 5 μm 4.6×150 mm column.

### General procedures for the synthesis of the IWR1 analogs.

To a solution of the bicycloalkanedicarboxylic acid monoester (1.0 mmol, 1.0 equiv) in methylene chloride (2 mL) was added triethylamine (0.42 mL, 3.0 mmol, 3.0 equiv) and HATU (456 mg, 1.2 mmol, 1.2 equiv). After stirring for 5 min, 8-aminoquinoline (159 mg, 1.1 mmol, 1.1 equiv) was added, and the reaction was monitored by TLC and LC-MS. Upon completion, water (2 mL) was added, and the organic layer was washed with hydrochloric acid (1.0 N, 2 mL ×2), saturated sodium bicarbonate (2 mL ×2) and brine (2 mL), dried over anhydrous sodium sulfate, and concentrated. The residue was then dissolved in a mixture of tetrahydrofuran (1 mL), methanol (0.5 mL) and water (0.5 mL). Lithium hydroxide monohydrate (126 mg, 3.0 mmol, 3.0 equiv) was then added, and the reaction was monitored by TLC and LC-MS. Upon completion, the solution was acidified by hydrochloric acid (1 N) to pH 5, concentrated, and then purified by flash column chromatography (silica gel, methanol/methylene chloride). To a solution of the resulting bicycloalkamic acid (1.0 mmol, 1.0 equiv) in *tert*-butanol (2.0 mL) was added triethylamine (0.56 mL, 4.0 mmol, 4.0 equiv) and diphenyl phosphoryl azide (0.32 mL, 1.5 mmol, 1.5 equiv). After stirring for 1 h, the solution was refluxed for 20 h before concentrated, redissolved in ethyl acetate (2 mL), washed with brine (2 mL ×2), dried over anhydrous sodium sulfate, concentrated, and purified by flash column chromatography (silica gel, ethyl acetate/hexanes). To a solution of the resulting Boc-aminoamide (1.0 mmol, 1.0 equiv) in methylene chloride (1.0 mL) was added trifluoroacetic acid (0.2 mL). After stirring for 1 h, the solution was concentrated and redissolved in dimethylformamide (2.0 mL) before triethylamine (0.28 mL, 2.0 mmol, 2.0 equiv), 4Å molecular sieves (2.0 g), and nadic anhydride (164 mg, 1.0 mmol, 1.0 equiv) were added. After stirring at 120 °C for 2 h, the solution was cooled to room temperature, filtered, diluted with ethyl acetate, washed with water (5 mL ×2) and brine (5 mL ×2), dried over anhydrous sodium sulfate, concentrated, and purified by flash column chromatography (silica gel, ethyl acetate/hexanes) to give the IWR compound.

### Bicyclo[1.1.1]pentamic acid 5.

90% yield over 2 steps; mp 246 °C; ^1^H NMR (400 MHz, CDCl_3_) δ 10.03 (s, 1H), 8.88 (dd, *J* = 4.4, 1.7 Hz, 1H), 8.78 (dd, *J* = 6.4, 2.7 Hz, 1H), 8.31–8.24 (m, 1H), 7.63–7.51 (m, 3H), 2.58 (s, 6H); MS (ESI) calculated for C_16_H_15_N_2_O_3_ (M+H)^+^ 283.1, found 283.2.

### (Boc-amino)bicyclo[1.1.1]pentamide 6.

61% yield; mp 69 °C; ^1^H NMR (400 MHz, CDCl_3_) δ 10.04 (s, 1H), 8.82 (dd, *J* = 4.3, 1.7 Hz, 1H), 8.76 (dd, *J* = 7.0, 2.1 Hz, 1H), 8.20 (dd, *J* = 8.3, 1.7 Hz, 1H), 7.60–7.46 (m, 3H), 2.49 (s, 6H), 1.48 (s, 9H); MS (ESI) calculated for C_20_H_24_N_3_O_3_ (M+H)^+^ 354.2, found 354.2.

### IWR1-BCP (2).

97% yield over 2 steps; mp 57 °C; ^1^H NMR (600 MHz, CD_3_CN) δ 9.94 (s, 1H), 8.87 (dd, *J* = 4.2, 1.6 Hz, 1H), 8.66 (dd, *J* = 7.6, 1.3 Hz, 1H), 8.30 (dd, *J* = 8.3, 1.7 Hz, 1H), 7.64–7.53 (m, 3H), 6.12 (t, *J* = 1.9 Hz, 2H), 3.28 (dp, *J* = 3.4, 1.6 Hz, 2H), 3.24 (dd, *J* = 3.0, 1.6 Hz, 2H), 2.58 (s, 6H), 1.63 (dt, *J* = 8.6, 1.7 Hz, 1H), 1.54 (dt, *J* = 8.5, 1.5 Hz, 1H); MS (ESI) calculated for C_24_H_22_N_3_O_3_ (M+H)^+^ 400.2, found 400.2.

### Bicyclo[2.1.1]hexamic acid 5a.

95% yield over 2 steps; mp 154 °C; ^1^H NMR (400 MHz, CD_3_OD) δ 8.82 (dd, *J* = 4.2, 1.7 Hz, 1H), 8.61 (dd, *J* = 7.6, 1.4 Hz, 1H), 8.25 (dd, *J* = 8.3, 1.7 Hz, 1H), 7.61–7.47 (m, 3H), 2.28–2.21 (m, 2H), 2.20–2.14 (m, 2H), 2.13–2.07 (m, 2H), 1.93–1.85 (m, 2H); MS (ESI) calculated for C_17_H_17_N_2_O_3_ (M+H)^+^ 297.1, found 297.2.

### (Boc-amino)bicyclo[2.1.1]hexamide 6a.

27% yield; mp 151 °C; ^1^H NMR (400 MHz, CDCl_3_) δ 10.08 (s, 1H), 8.82 (ddd, *J* = 9.0, 5.8, 1.8 Hz, 2H), 8.23 (dd, *J* = 8.3, 1.7 Hz, 1H), 7.61–7.47 (m, 3H), 2.40 (s, 2H), 2.19–2.10 (m, 2H), 2.03–1.94 (m, 4H), 1.48 (s, 9H); MS (ESI) calculated for C_21_H_26_N_3_O_3_ (M+H)^+^ 368.2, found 368.2.

### IWR-BCHx (9).

49% yield over 2 steps; mp 152 °C; ^1^H NMR (600 MHz, CD_3_CN) δ 9.98 (s, 1H), 8.86 (dd, *J* = 4.2, 1.7 Hz, 1H), 8.71 (dd, *J* = 7.5, 1.4 Hz, 1H), 8.31 (dd, *J* = 8.3, 1.7 Hz, 1H), 7.64–7.53 (m, 3H), 6.14 (t, *J* = 1.9 Hz, 2H), 3.27 (dq, *J* = 3.4, 1.7 Hz, 2H), 3.23 (dd, *J* = 3.0, 1.6 Hz, 2H), 2.28 (dd, *J* = 4.2, 1.8 Hz, 2H), 2.14–2.05 (m, 6H), 1.62 (dt, *J* = 8.5, 1.8 Hz, 1H), 1.53 (dt, *J* = 8.7, 1.6 Hz, 1H); MS (ESI) calculated for C_25_H_24_N_3_O_3_ (M+H)^+^ 414.2, found 414.2.

### Bicyclo[2.2.1]heptamic acid 5b.

93% yield over 2 steps; mp 169 °C; ^1^H NMR (400 MHz, CDCl_3_) δ 10.12 (s, 1H), 8.82 (dd, *J* = 4.3, 1.7 Hz, 1H), 8.79 (dd, *J* = 7.2, 1.8 Hz, 1H), 8.18 (dd, *J* = 8.3, 1.7 Hz, 1H), 7.59–7.44 (m, 3H), 2.30–2.22 (m, 4H), 2.22–2.17 (m, 2H), 2.04–1.95 (m, 2H), 1.91–1.82 (m, 2H); MS (ESI) calculated for C_18_H_19_N_2_O_3_ (M+H)^+^ 311.1, found 311.2.

### (Boc-amino)bicyclo[2.2.1]heptamide 6b.

14% yield; mp 135 °C; ^1^H NMR (400 MHz, CDCl_3_) δ 10.08 (s, 1H), 8.86 (dd, *J* = 4.4, 1.7 Hz, 1H), 8.81 (dd, *J* = 7.3, 1.7 Hz, 1H), 8.33–8.26 (m, 1H), 7.66–7.50 (m, 3H), 2.34–2.16 (m, 4H), 2.15–1.94 (m, 4H), 1.92–1.80 (m, 2H), 1.47 (s, 9H); MS (ESI) calculated for C_22_H_28_N_3_O_3_ (M+H)^+^ 382.2, found 382.2.

### IWR1-BCHp (10).

37% yield over 2 steps; mp 164 °C; ^1^H NMR (600 MHz, CD_3_CN) δ 10.02 (s, 1H), 8.86 (dd, *J* = 4.2, 1.7 Hz, 1H), 8.72 (dd, *J* = 7.5, 1.4 Hz, 1H), 8.31 (dd, *J* = 8.2, 1.7 Hz, 1H), 7.62–7.53 (m, 3H), 6.12 (t, *J* = 1.9 Hz, 2H), 3.27 (dq, *J* = 3.4, 1.6 Hz, 2H), 3.20 (dd, *J* = 3.1, 1.6 Hz, 2H), 2.52–2.47 (m, 2H), 2.42–2.34 (m, 2H), 2.11–2.02 (m, 2H), 1.83 (tt, *J* = 9.1, 4.4 Hz, 2H), 1.71 (tdd, *J* = 11.6, 5.8, 3.5 Hz, 2H), 1.61 (dt, *J* = 8.5, 1.8 Hz, 1H), 1.52 (dt, *J* = 8.6, 1.7 Hz, 1H); MS (ESI) calculated for C_26_H_26_N_3_O_3_ (M+H)^+^ 428.2, found 428.2.

### Bicyclo[2.2.2]octamic acid 5c.

87% yield over 2 steps; mp 239 °C; ^1^H NMR (400 MHz, CDCl_3_) δ 10.16 (s, 1H), 8.83 (dd, *J* = 4.2, 1.7 Hz, 1H), 8.77 (dd, *J* = 7.1, 1.9 Hz, 1H), 8.17 (dd, *J* = 8.3, 1.7 Hz, 1H), 7.57–7.44 (m, 3H), 2.10–2.03 (m, 6H), 2.02–1.94 (m, 6H); MS (ESI) calculated for C_19_H_21_N_2_O_3_ (M+H)^+^ 325.2, found 325.2.

### (Boc-amino)bicyclo[2.2.2]octamide 6c.

7% yield; mp 67 °C; ^1^H NMR (400 MHz, CDCl_3_) δ 10.13 (s, 1H), 8.84 (dd, *J* = 4.3, 1.7 Hz, 1H), 8.76 (dd, *J* = 7.2, 1.8 Hz, 1H), 8.21 (dd, *J* = 8.2, 1.7 Hz, 1H), 7.60–7.46 (m, 3H), 2.16–2.09 (m, 6H), 2.02–1.94 (m, 6H), 1.44 (s, 9H); MS (ESI) calculated for C_23_H_30_N_3_O_3_ (M+H)^+^ 396.2, found 396.2.

### IWR1-BCO (8).

7% yield over 2 steps; mp 188 °C; ^1^H NMR (600 MHz, CD_3_CN) δ 10.08 (s, 1H), 8.85 (dd, *J* = 4.2, 1.7 Hz, 1H), 8.68 (dd, *J* = 7.6, 1.4 Hz, 1H), 8.30 (dd, *J* = 8.3, 1.7 Hz, 1H), 7.63–7.51 (m, 3H), 6.10 (t, *J* = 1.9 Hz, 2H), 3.24 (dq, *J* = 3.4, 1.8 Hz, 2H), 3.11 (dd, *J* = 3.0, 1.6 Hz, 2H), 2.30–2.23 (m, 7H), 2.04–1.99 (m, 7H); MS (ESI) calculated for C_27_H_28_N_3_O_3_ (M+H)^+^ 442.2, found 442.2.

### Computation.

The computations were performed on Spartan’18 suite of programs using DFT/6–311G+(d,p) for the equilibrium geometries and single-point energies, and SMD/6–31G(d) for the solvation energies. Docking was performed on AutoDock Vina using PDB 4DVI.

## Supplementary Material

supporting information
