# Supplementary material for: Exceptional reactivity of the bridgehead amine on bicyclo[1.1.1]pentane
Source: ARKIVOC. Author manuscript; Available in PMC 2023 Oct 2. (PMC10544781; doi:10.24820/ark.5550190.p012.003)
Supplement: supporting information [file NIHMS1926680-supplement-supporting_information.pdf]

Supplementary Material

Exceptional reactivity of the bridgehead amine on bicyclo[1.1.1]pentane

Yong Lu and Chuo Chen*

Department of Biochemistry, UT Southwestern Medical Center
5323 Harry Hines Boulevard, Dallas, TX 75390-9038
Email: Chuo.Chen@UTSouthwestern.edu

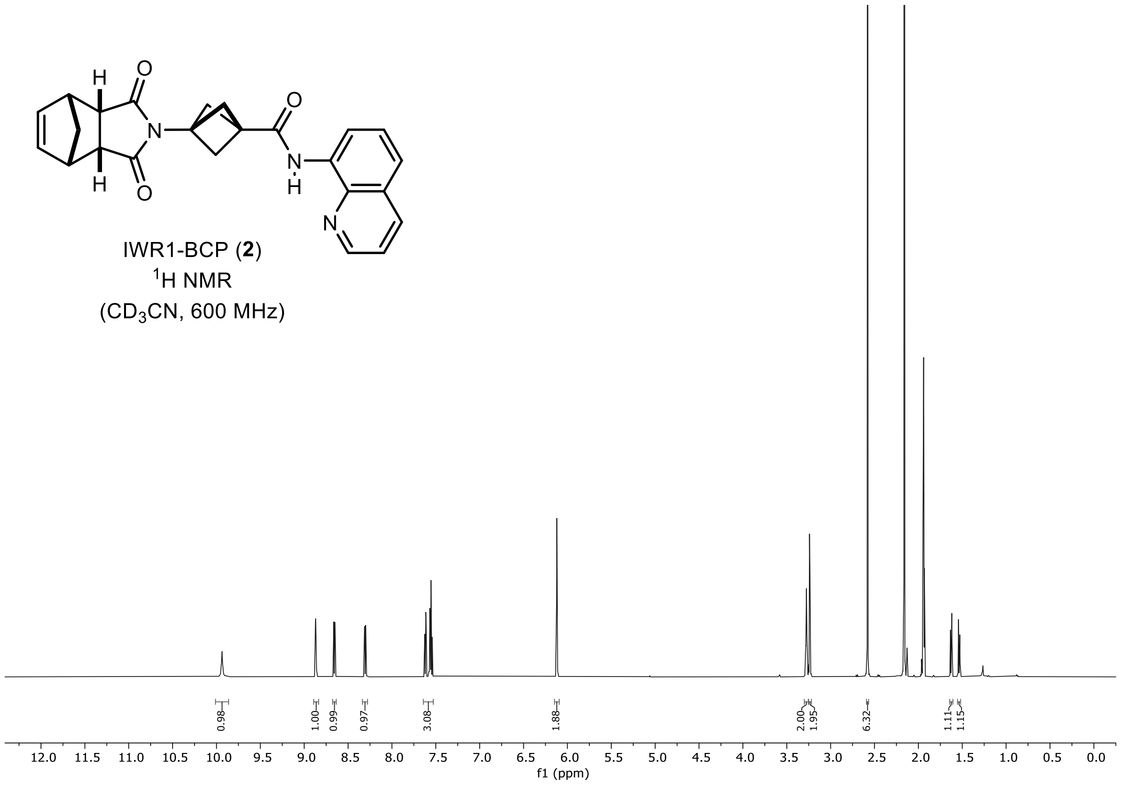
H
O
N
O
H
O
N
H
N
IWR1-BCP (**2**)
^{1}H NMR
(CD_3CN, 600 MHz)
0.98
1.00
0.99
0.97
3.08
1.88
2.00
1.95
6.32
1.11
1.15
12.0 11.5 11.0 10.5 10.0 9.5 9.0 8.5 8.0 7.5 7.0 6.5 6.0 5.5 5.0 4.5 4.0 3.5 3.0 2.5 2.0 1.5 1.0 0.5 0.0
f1 (ppm)

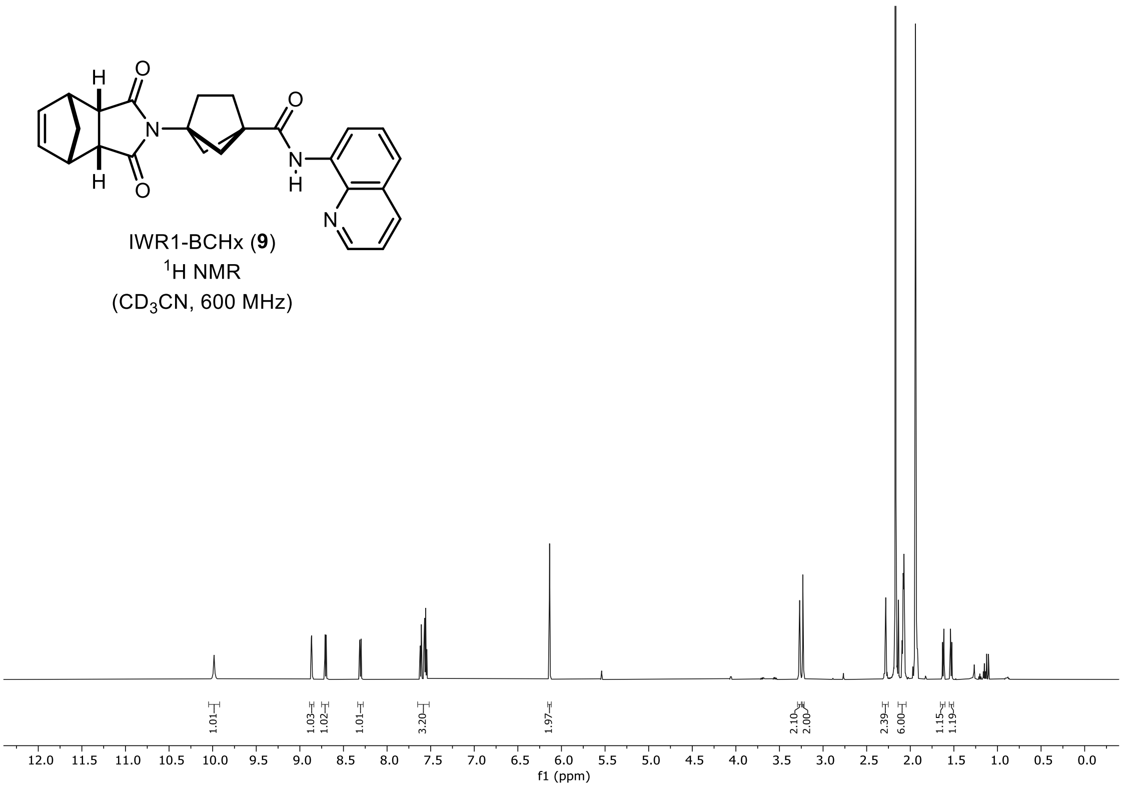
H
O
N
O
H
O
N
H
N
IWR1-BCHx (**9**)
^{1}H NMR
(CD_3CN, 600 MHz)
1.01
1.03
1.02
1.01
3.20
1.97
2.10
2.00
2.39
6.00
1.15
1.19
12.0 11.5 11.0 10.5 10.0 9.5 9.0 8.5 8.0 7.5 7.0 6.5 6.0 5.5 5.0 4.5 4.0 3.5 3.0 2.5 2.0 1.5 1.0 0.5 0.0
f1 (ppm)

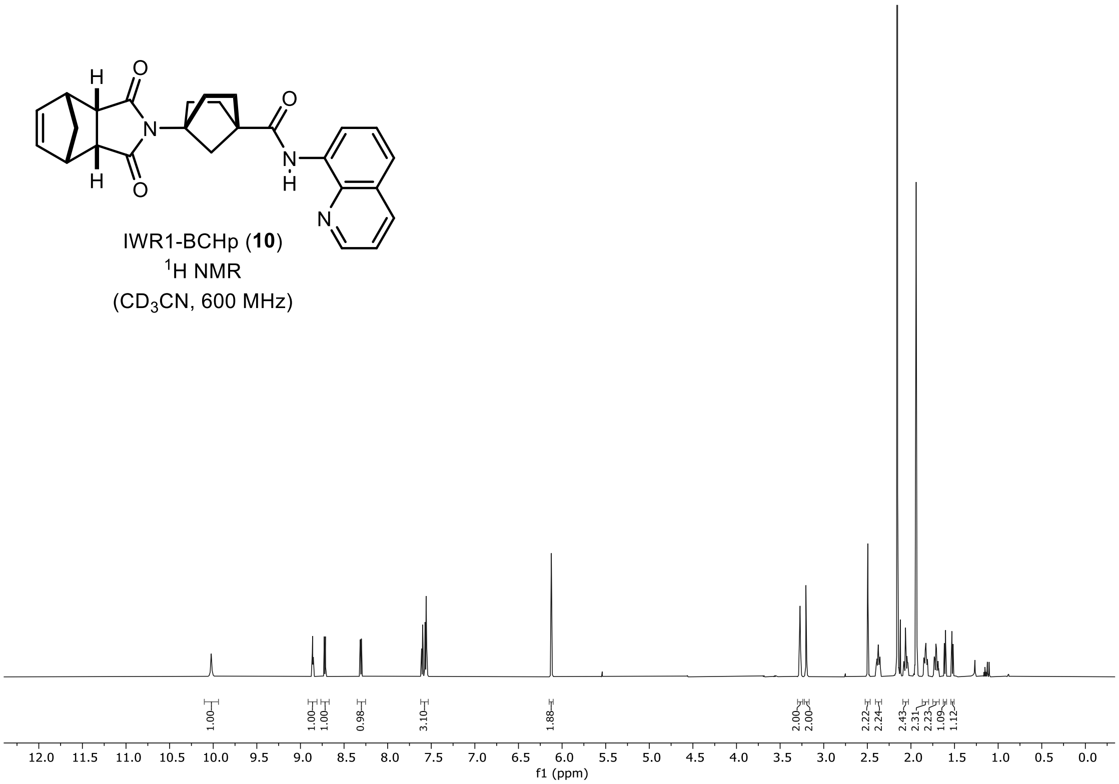
IWR1-BCHp (**10**)
^{1}H NMR
(CD_3CN, 600 MHz)
f1 (ppm)

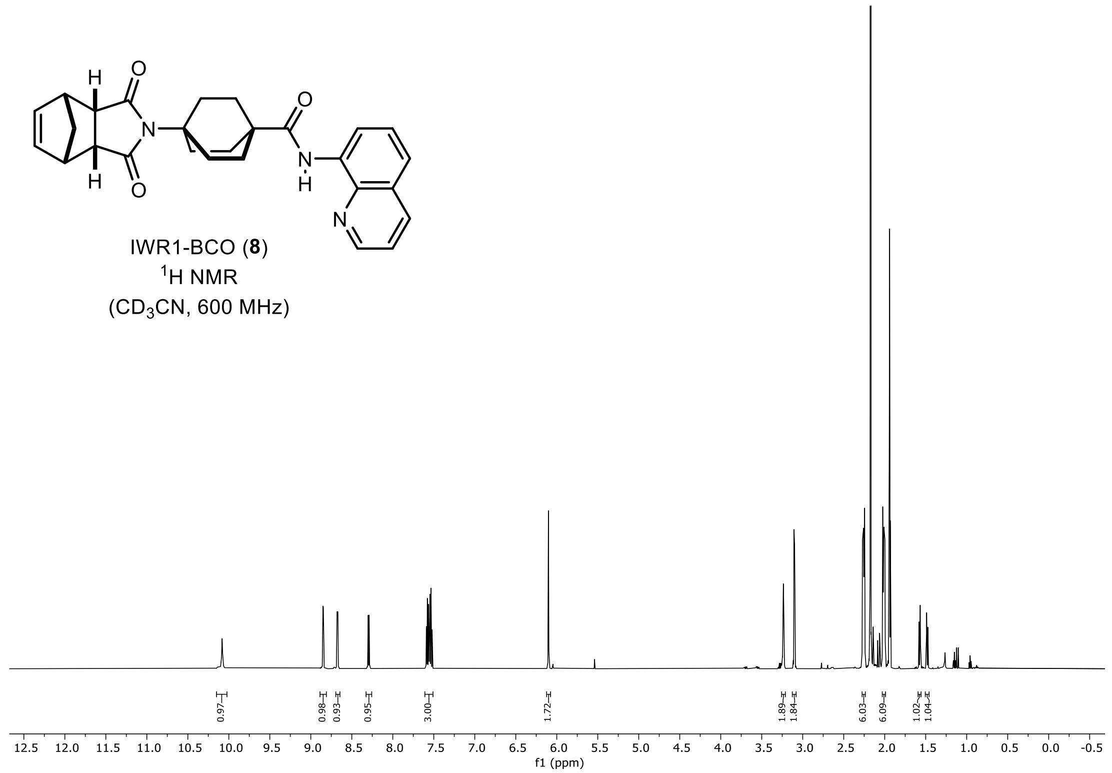
IWR1-BCO (**8**)
^{1}H NMR
(CD_3CN, 600 MHz)
f1 (ppm)
